# Establishing a treatment protocol for concomitant major burn and trauma patients: a tropical Asian hospital’s experience

**DOI:** 10.1186/s41038-017-0088-6

**Published:** 2017-07-05

**Authors:** Yee Onn Kok, Nadia Sim, Priya Tiwari, Ting Hway Wong, Si Jack Chong

**Affiliations:** 1 Department of Plastic, Reconstructive and Aesthetic Surgery, Singapore General Hospital, Level 5, Academia, Singapore, 169865 Singapore; 2 Yong Loo Lin School of Medicine, National University of Singapore, 1E Kent Ridge Road, Level 11, NUHS Tower Block, Singapore, 119228 Singapore; 3 Division of Plastic, Reconstructive and Aesthetic Surgery, Department of Surgery, National University Health System, Level 8, NUHS Tower Block, 1E Kent Ridge Road, Singapore, 119228 Singapore; 4 Department of General Surgery, Singapore General Hospital, Level 5, Academia, Singapore, 169865 Singapore

**Keywords:** Burns, Trauma, Protocol

Dear Editor,

Burns and trauma represent two of the most severe injuries that both developing and developed countries face today. Concomitant burn and traumatic injuries are significant in terms of disease burden and cost of treatment due to associated high morbidity and mortality.

Existing literature for burn and traumatic injuries encompass injuries derived from terrorism, warfare, natural disasters [1], and accidents. The most significant to date is the large-scale study by Kalson et al. on burn and traumatic injuries in England and Wales [2]. However, literature and outcomes regarding the multidisciplinary management and approach to concomitant burns and trauma in a developed Asian center are limited.

The Singapore General Hospital (SGH) Burns Centre is the only adult burn center in Singapore and is the regional referral center in Southeast Asia. Our center receives 93% of all burn patients in Singapore including mass casualties from the region [3].

We conducted a retrospective case-control “trauma/burns” study of 11 patients with concomitant major burn and traumatic injuries at SGH from 1998 to 2012 obtained from the SGH Burns Registry. Inclusion criteria were trauma with multiple injuries requiring activation of the trauma team (e.g., intracranial injuries, long bone fractures, abdominal injuries, inhalational injuries) and major burns of more than 15% total body surface area (TBSA). Exclusion criteria were patients who were dead on arrival or repatriation cases. We compared this group to a control group of 11 major burn (>15% TBSA) patients without traumatic injuries admitted during the same time period. Controls were randomly matched for TBSA and age.

Both groups had similar demographics and burn characteristics for comparison. The median age is 37 and 39 years in the trauma/burns group and the control group, respectively. Majority of the patients are males (90.9% (*n* = 10) in trauma/burns, 81.8% (*n* = 9) in control). The median TBSA is 32% for both groups (16–90% and 15–88% for the trauma/burns group and control group, respectively) and of deep dermal depth.

Occupational burns (e.g., electric and flash explosions, generator and gas cylinder explosions) is the majority in both study and control groups (73% (*n* = 8) of patients from both groups). This highlights the importance of workplace safety such as protective equipment, regular preventive maintenance and job-specific hazard analysis implementation.

In our study, the severity and high mortality (36.4%) of patients with concomitant burn and traumatic injuries highlights the focus on managing these critically ill patients well. In contrast, the mortality for control group is 9.1% (*n* = 1) and the international average mortality rate is lower for burn injuries alone (3.1–18%) [4, 5]. Burn intensive care unit (BICU) admission rate was 90.9% (*n* = 10) in trauma/burn patients (due to their severe injuries) and 45.5% (*n* = 5) in the control burns only group. Patients in the trauma/burns group mostly belonged to Clavien Dindo Classification terminal grades of 4A (*n* = 7) and 5 (*n* = 4) compared to patients in the control group who were mainly grades of 3B (*n* = 6) and 3Bd (*n* = 4).

A seasoned protocol, early resuscitation, robust burn care within the golden hour and definitive surgeries done within 24 h is important for survival. The median time of injury to admission (inclusive of time needed for transfer) was 1 h (0.2–48 h) and 2.5 h (0.25–144 h) in the trauma/burns and control group, respectively. Singapore’s small geographical area ensured that all local trauma/burn patients arrived almost within 1 h of injury. Early resuscitation and communication between paramedics and the emergency department allows for standby of the trauma and burns teams. As such, none of the trauma/burns patients passed away within 24 h of resuscitation.

None of the concomitant trauma that patients in the trauma/burns group suffered was immediately life threatening such as intrathoracic and intra-abdominal injuries. Rather, it was often severe inhalational injury coupled with the systemic inflammatory response syndrome (SIRS) overdrive from the trauma and burns double hit that precipitated the “early mortalities” (days 3–8) and multi-organ failure. Three out of four mortalities were secondary to respiratory failure from acute respiratory distress syndrome (*n* = 1) and severe inhalational burns (*n* = 2).

Sepsis typically sets in from the third day onwards of the burns injury. The rate of mortality is high (between 19.5 and 23%) amongst burn patients with sepsis-related complications. Patients with severe burn injuries are exceptionally vulnerable to infections due to large wounds, frequent invasive procedures such as artificial ventilation, tracheotomy, and arterial lines and renal replacement therapy. Hence, early prediction of sepsis onset, timely intervention, and prevention can lower mortality and morbidity.

Median overall length of stay was 41 days (2–68 days) in trauma/burns patients compared to 16 days (3–52 days) for the control burns only group. This prolonged hospitalization is a predictor of prolonged SIRS and also the possibility of compensatory anti inflammatory response syndrome (CAIRS) [6]. Longer durations of hospitalization also predispose patients to high mortality nosocomial infections such as multi-drug resistant *Acinetobacter baumannii* and *Pseudomonas* infections [7].

All trauma/burns mortalities required dialysis for acute renal failure and varying severities of inhalational injuries and respiratory failure. The American Burn Association and National Burn Repository recognizes the presence of inhalational injury as a major predictor of mortality [8]. Pre-renal failures from inadequate or delayed fluid resuscitation, sepsis, rhabdomyolysis, and nephrotoxic antibiotics or medications also cause multiple hits to the kidney. All patients who had acute renal failure requiring dialysis passed away. Early identification and management of renal failure in patients with concomitant burn and traumatic injuries is imperative in preventing further complications and mortality.

In summary, golden hour early resuscitation with the pre-hospital team, aggressive dialysis for oliguria, early intubation with cervical clearance, sepsis prevention and surgery within 24 h with early postoperative rehabilitation, and psychosocial support are hallmarks of our hospital protocol. Nonetheless, mortality is still high.

More resources and healthcare planning and policy should be carefully considered to create the best setup for expedient multidisciplinary care. We have recently set up a combined national burns and trauma database that will facilitate review and formulation of evidence-based practice in the management of patients with concomitant burns and traumatic injuries.

Trauma and burn patients highlight the dire need to optimize outcomes through pre-hospital education, prevention along with a streamlined hospital resuscitation protocol and timely surgery.

## Abbreviations

BICU: Burn intensive care unit
CAIRS: Compensatory anti inflammatory reponse syndrome
SGH: Singapore general hospital
SIRS: Systemic inflammatory response syndrome
TBSA: Total burn surface area
